# *Mink* S38G Gene Polymorphism and Atrial Fibrillation in the Chinese Population: A Meta-Analysis of 1871 Participants

**DOI:** 10.1155/2014/768681

**Published:** 2014-02-16

**Authors:** Yan-yan Li, Lian-sheng Wang, Xin-zheng Lu

**Affiliations:** ^1^Department of Geriatrics, First Affiliated Hospital of Nanjing Medical University, Nanjing 210029, China; ^2^Department of Cardiology, First Affiliated Hospital of Nanjing Medical University, Nanjing 210029, China

## Abstract

*Mink* gene S38G polymorphism in the **β**-subunit of slow activating component of the delayed rectifier potassium channel current potassium channel has been associated with increased atrial fibrillation (AF) risk. However, the individual studies results were still controversial. To investigate the association of *Mink* S38G gene polymorphisms with AF, a meta-analysis including 1871 subjects from six individual studies was conducted. *Mink* S38G gene polymorphism was significantly related to AF under allelic (OR: 1.380, 95% CI: 1.200–1.600, *P* < 0.00001), recessive (OR: 1.193, 95% CI: 1.033–1.377, *P* = 0.017), dominant (OR: 1.057, 95% CI: 1.025–1.089, *P* < 0.00001), additive (OR: 1.105, 95% CI: 1.036–1.178, *P* = 0.002), homozygous (OR: 1.128, 95% CI: 1.068–1.191, *P* < 0.00001), and heterozygous genetic models (OR: 1.078, 95% CI: 1.014–1.146, *P* = 0.016). A significant association between *Mink* S38G gene polymorphism and AF risk was found. G allele carriers may predispose to AF.

## 1. Introduction

Atrial fibrillation (AF) is the most common arrhythmia in clinical practice [1]. Its prevalence varies among countries, with different ranges in community- and hospital-based studies (0.1–4% and 2.8–14%, resp.). The incidence of AF increases with age, ranging from 1% in youth to approximately 10% above the age of 80 [2]. AF can cause serious complications such as stroke and congestive heart failure [3, 4]; however, its pathogenesis is still unclear. Electricity remodeling has an important effect on the AF process [5]. The electrophysiologic basis of electricity remodeling is characterized by ion channel dysfunction as the intracellular calcium overloads and the internal flow of L-type calcium channel decreases [6]. The increase of the slow activating component of the delayed rectifier potassium channel current (Iks) can relatively shorten the phase 2 plateau of the action potential and the refractory period which lead to AF occurrence. The myocardium Iks *β*-subunit gene (KCNE1,* Mink*) S38G gene polymorphism has been associated with Iks function enhancement [7].

*Mink *gene is located in 21q22.1-22.2, spans about 390 bp, encodes 130 amino acids, and constitutes the Iks *β*-subunit. Lai et al. (2002) found that a *Mink *gene rs1805127 locus at the 112th base adenine (A) was substituted with a guanine (G) at the exon region, resulting in the wild-type Serine (Ser, S) being replaced by Glycine (Gly, G) at 38th amino acid in the 3rd exon. They also found that the *Mink* S38G gene polymorphism was correlated with AF [7].

Several studies have associated *Mink* S38G gene polymorphism with AF; however, the results were contradictory. Zeng et al. (2005) found that *Mink* S38G gene polymorphism was not associated with AF in a study conducted with Beijing Chinese population [8]. By contrast, Qu and Yin found that *Mink* 38G allele was significantly correlated with AF in a study conducted with Chongqing Chinese population [9].

In the current study, a meta-analysis that includes 856 AF patients and 1015 controls was conducted to determine the relationship of *Mink* S38G gene polymorphism with AF in the Chinese population.

## 2. Materials and Methods

### 2.1. Publication Search and Inclusion Criteria

The terms “*Mink*,” “S38G,” “*KCNE1*,” “atrial fibrillation,” and “polymorphism” were searched in the electronic databases of PubMed, Embase, Web of Science, China Biological Medicine Database, and China National Knowledge Infrastructure. The last updated research date was November 12, 2013, and the publication years ranged from 2002 to 2011.

The selected studies have to be consistent with the following major criteria: (a) assessment of the *Mink* S38G gene polymorphism and nonvalvular AF, studies on the family history of AF or valvular AF would be excluded; (b) AF was diagnosed as the episodes ≥ 2 occasions (>6 months apart) by serial 12-lead electrocardiography (ECG) or 24 h Holter monitoring. The ECG diagnosis criteria were as follows: (1) *P* wave disappearance and replacement with irregular baseline fluctuation, with a frequency ranging from 350 per minute to 600 per minute, namely, *f* wave; (2) extremely irregular ventricular rate; (3) inconsistent QRS complex morphology, the QRS complex becomes broader and changes in the presence of aberrant ventricular conduction and the RR internal remains extremely irregular; (c) the study should be a case-control or cohort study published in an official journal; (d) the study should conform to the Hardy-Weinberg equilibrium (HWE).

### 2.2. Data Extraction

The data were extracted according to a standard protocol. Three investigators carried out the meta-analysis; two of whom searched for parallel studies, and the third served as the arbitrator in case of discordance between the two investigators. Studies that failed to meet the inclusion criteria, were published repeatedly, or provided insufficient data were excluded. Similar data from different studies were only used once. The abstracted data contained the following: the first author's name, publication year, study region, PubMed identifier number, study design, case selection method, matching factors (if applicable), source population, mean ages of the cases and controls, sample size, variants, major and minor alleles, genotype counts for the cases and controls, HWE among controls, and genotyping methods used. The six selected studies were scored based on the criteria implemented from published recommendations on the evaluation of the quality of genetic association studies [13].

### 2.3. Statistical Analyses

In the present meta-analysis, six genetic models, including the allelic (distribution of G allelic frequency of *Mink* S38G gene polymorphism), recessive (GG versus SG + SS), dominant (GG + SG versus SS), additive (G versus S), homozygous (GG versus SS), and heterozygous genetic models (SG versus SS), were adopted. The relationship of* Mink* S38G gene polymorphism with AF was compared using odds ratio (OR) and its corresponding 95% confidence interval (CI). The Chi-square-based *Q*-tests were used to calculate the heterogeneity among the individual studies, with significance level at *P* < 0.10 [14]. The random-effect model was used to estimate the pooled OR (DerSimonian and Laird methods) when heterogeneity existed among the individual studies [15]. Otherwise, the fixed-effect model was used (the Mantel-Haenszel method) [16]. The pooled OR was determined by *Z*-test, with significance level at *P* < 0.05.

HWE was assessed using Fisher's exact test, with significance level at *P* < 0.05. The funnel plot was used to estimate the potential publication bias. Egger's linear regression test on the natural logarithm scale of the OR was used to assess the funnel plot asymmetry, with significance level at *P* < 0.05 [17]. STATA 11.0 was used to perform the statistical analyses (StataCorp, College Station, TX, USA). The “Venice criteria” were applied to each statistically significant association in the current meta-analysis to assess the credibility of the evidence [18].

## 3. Results

### 3.1. Studies and Populations

Nineteen studies were retrieved, among which six studies conformed to the inclusion criteria. Among the 13 excluded studies, five were reviews and five studies were not involved in *Mink* S38G gene polymorphism or AF. One study was excluded for being against the HWE. Two studies associated with *Mink* S38G gene polymorphism and AF were performed in non-Chinese population [19, 20]. Following the 10-point scoring sheet by Clark and Baudouin, all selected studies achieved a score of no less than 8 [13]. The data were extracted from 856 cases and 1015 controls (Table 1) [7–9, 18–20].

### 3.2. Pooled Analyses

**Figure 1:**
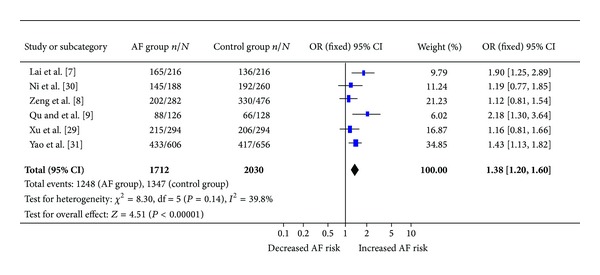
Forest plot of AF associated with *Mink* S38G gene polymorphism under an allelic genetic model (distribution of G allelic frequency of *Mink* S38G gene polymorphism) in the Chinese population.

*Mink* S38G gene polymorphism was significantly related to AF under allelic (OR: 1.380, 95% CI: 1.200–1.600, *P* < 0.00001), recessive (OR: 1.193, 95% CI: 1.033–1.377, *P* = 0.017), dominant (OR: 1.057, 95% CI: 1.025–1.089, *P* < 0.00001), additive (OR: 1.105, 95% CI: 1.036–1.178, *P* = 0.002), homozygous (OR: 1.128, 95% CI: 1.068–1.191, *P* < 0.00001), and heterozygous genetic models (OR: 1.078, 95% CI: 1.014–1.146, *P* = 0.016) (Table 2, Figure 1).

Heterogeneity was significant under the recessive (*P*_heterogeneity_ = 0.057, *I* ^2^ = 53.5%) and additive genetic models (*P*_heterogeneity_ = 0.066, *I* ^2^ = 51.6%). Metaregression was performed to investigate the heterogeneity source. Heterogeneity under the recessive model can be explained by the SS genotype number of the AF group (SS1, *P* = 0.003) and the SG genotype number of the AF group (SG1, *P* = 0.003). According to SG1, the whole population was divided into two subgroups. The studies with SG1 < 60 belonged to subgroup  1; the remaining studies with SG1 ≥ 60 belonged to subgroup  2. In the subgroup analysis stratified by SG1, AF risk was only significantly increased in subgroup 2 (*P* = 0.010) and in subgroup 1, no significant association between Mink S38G and AF was detected (*P* = 0.073). However, the association strength was much weaker in subgroup 2 (OR: 1.162, 95% CI: 1.036–1.302, *P* = 0.010) and the heterogeneity did not exist in subgroup 2 (*P*_heterogeneity_ = 0.106, *I* ^2^ = 55.4%). By contrast, the heterogeneity was enhanced in subgroup 1 (*P*_heterogeneity_ = 0.055, *I* ^2^ = 65.6%) (Tables 2-3, Figure 1).

The heterogeneity under the additive genetic model can also be explained by SS1 (*P* = 0.004) and SG1 (*P* = 0.003). The whole population was separated into two subgroups according to SG1, mentioned above. In the subgroup analysis stratified by SG1, AF risk increased significantly in both subgroups. However, the heterogeneity was distinctly reduced in subgroup 2 (*P*_heterogeneity_ = 0.316, *I* ^2^ = 13.1%), but still existed in subgroup 1 (*P*_heterogeneity_ = 0.038, *I* ^2^ = 69.4%) (Tables 2 and 4).

### 3.3. Bias Diagnostics

**Figure 2:**
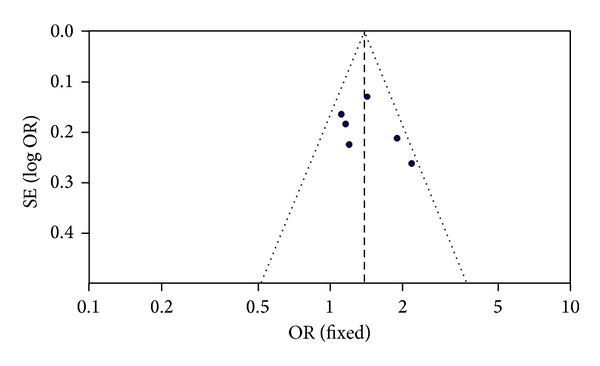
Funnel plot for studies of AF associated with *Mink* S38G gene polymorphism under an allelic genetic model (distribution of G allelic frequency of *Mink* S38G gene polymorphism) in the Chinese population. The horizontal and vertical axis correspond to the OR and confidence limits. OR: odds ratio; SE: standard error.

The funnel plot and Egger's test were used to evaluate the publication bias of the individual studies. A visual publication bias can be detected in the funnel plot (Figure 2). Egger's test confirmed a significant difference between the individual studies. Thus, significant publication bias existed in the present meta-analysis (allelic genetic model, *T* = −3.83, *P* = 0.019). The fail-safe number value was 40 under the additive genetic model which implied that 40 “null” studies should be added to render the general effect insignificant.

### 3.4. Sensitivity Analysis

**Figure 3:**
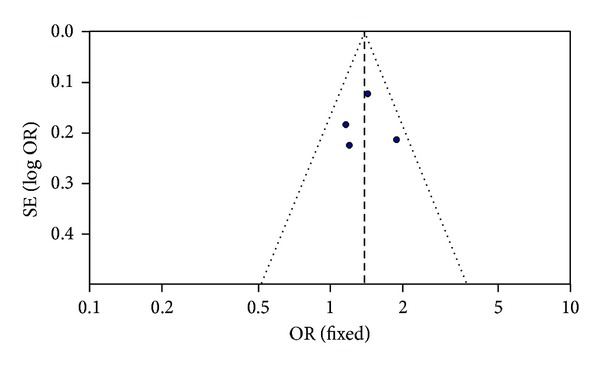
Funnel plot for studies of AF associated with *Mink* S38G gene polymorphism under an allelic genetic model (distribution of G allelic frequency of *Mink* S38G gene polymorphism) after sensitivity analysis in the Chinese population. The horizontal and vertical axis correspond to the OR and confidence limits. OR: odds ratio; SE: standard error.

A possible influence may be observed from the studies of Zeng et al. [8] and Qu and Yin [9]. Sensitivity analysis was conducted to assess the publication bias resource. After the two above studies were excluded, no obvious publication bias was shown in Egger's test (allelic genetic model, *T* = −3.31, *P* = 0.080) and the funnel plot was approximately symmetrical (Figure 3).

## 4. Discussion

In the present meta-analysis, *Mink* S38G gene polymorphism was significantly related to AF under allelic (OR: 1.380), recessive (OR: 1.193), dominant (OR: 1.057), additive (OR: 1.105), homozygous (OR: 1.128), and heterozygous genetic models (OR: 1.078) in the Chinese population. The current results suggested that the individuals with G allele of *Mink* S38G gene polymorphism may have higher AF susceptibility than the others in the Chinese population.

Heterogeneity existed under the recessive, additive, and multiplicative genetic models. Thus, subsequent meta-regression was performed to explore the heterogeneity source. Under the recessive model, confounding factors such as SS1 (*P* = 0.003) and SG1 (*P* = 0.003) were demonstrated to possibly explain the heterogeneity source. After adjusting SG1, the AF risk only increased significantly in subgroup 2 which was not detected in subgroup 1. Heterogeneity disappeared in subgroup 2, but still existed in subgroup 1, suggesting that SG1 was the primary heterogeneity source. Similarly, under the additive model, SS1 (*P* = 0.004) and SG1 (*P* = 0.003) were the heterogeneity sources. In the subgroup analysis adjusted by SG1, the heterogeneity disappeared in subgroup 2 but remained in subgroup 1, indicating that SG1 was the main heterogeneity source.

In the present study, the significant associations of *Mink* S38G variants with AF were assessed using five genetic models, including allelic, recessive, dominant, additive, homozygous, and heterozygous genetic models. The *Z*-test was used to calculate the *P* value; *P* < 0.05 suggested a significant association. The fixed model was used under the allelic and dominant genetic models for the reason that their *P*_heterogeneity_ > 0.10. Since the *P*_heterogeneity_ < 0.10 under the recessive and additive genetic models, the random model was adopted.

The exclusion and inclusion criteria were addressed in detail and were strictly followed in the current meta-analysis. Based on the score system by Clark and Baudouin [13], all selected studies were with high quality. In the present meta-analysis, all individual studies were case-control studies published in official journals, where AF diagnostic was inconsistent with the major inclusion criteria. Family AF history has been excluded; thus, the meta-analysis results were objective and credible. Other unpublished studies that may contribute to the publication bias were not included in the current meta-analysis. Similarly, the relatively small sample size from the published studies also led to possible publication bias and heterogeneity among the individual studies. The sensitivity analysis showed that publication bias may come from the studies of Zeng et al. [8] and Qu and Yin [9]. After these studies were excluded, the publication bias disappeared. Based on the Venice criteria by Ioannidis et al., the epidemiology credibility was strong in all of the five genetic models [18].

AF is one of the most common clinical arrhythmias with complex pathogenesis. Recent studies showed that AF attack has a hereditary susceptibility. The genome-wide association studies (GWAS) identified three distinct genetic loci on chromosomes 1q21, 4q25, and 16q22 that are associated with AF. Susceptibility loci in 3p22, 5q35, 7q31, 12p12, and 12q24 were also identified by GWAS for PR interval duration. Only one locus around the gene NOS1AP was identified in the QT interval, another cardiovascular trait that is also a good indicator for AF [21].

At present, the gene variation encoding the ion channel was the pathologic factor for familial AF that reduced the Iks [22]. The Iks channel *α* and *β* subunits were encoded by *KCNQ1* and *Mink* genes, respectively [23]. On the physiological status, cardiac Iks channel participates in the atrial repolarization, especially on the terminal stage of action potential associated with frequency dependence action potential time interval shortening and atrial tissue electricity remodeling. *Mink* gene mutations can lead to various malignant arrhythmias such as familial long QT syndrome (LQT_S_) and Jervell syndrome [24, 25]. Moreover, the *Mink* gene polymorphisms were associated with drug-induced LQT_S_ and nonfamilial arrhythmias [26]. Chevillard et al. (1993) found that the *Mink* gene mRNA expression increased distinctly in the atrial tissue of the AF patients [27]. Chen et al. (2003) found that the interaction of Mink protein with other proteins formed the arrhythmia matrix that can strike the onset or maintenance of AF [28]. Thus, the *Mink* gene is important in regulating cardiac rhythm. The possible reasons for the *Mink* 38G allele associated with AF were as follows. (a) *Mink* 38G allele could increase *Mink* gene expression and Iks channel protein synthesis. (b) *Mink* 38G allele could increase Iks current, thereby shorten the action potential duration, and be predisposed to AF [9].

Lin et al. (2009) performed a meta-analysis to explore *Mink* S38G gene polymorphism and AF and found that the GG genotype was significantly associated with increased AF risk [29]. However, their research was associated with some defects. First, two studies deviating from HWE were not excluded from their study [30, 31]. Second, two studies published in 2008 were not included in their meta-analysis [9, 19]. Compared with their work, the abovementioned four studies were appropriately addressed in the current meta-analysis. In addition, the study published in 2011 was also retrieved and supplemented in the present study [11]. Therefore, the current results should be more objective and credible than Zhang's work.

However, limitations still existed in the present meta-analysis. Large-scale studies on the association of AF with *Mink* S38G gene polymorphism were still relatively insufficient. The relatively large effect sizes in the current meta-analysis may be attributed to the limited sample size. The Mink expression level was influenced by the *Mink* S38G gene polymorphism and by other gene polymorphisms, such as KCNQ1 S140G, KCNE2 R27C, and KCNE3 R53H mutations.

Therefore, *Mink* S38G gene polymorphism was significantly associated with AF susceptibility. Patients with the G allele may be predisposed to AF. The current conclusion may guide the formulation of new individual AF diagnosis parameters and therapeutic strategies. Considering the abovementioned limitations, more large-scale studies are needed to confirm the above conclusion.

## Figures and Tables

**Table 1:** Characteristics of the investigated studies of the association of *Mink* S38G gene polymorphism and atrial fibrillation (AF) in the Chinese population.

Author	Year	AF			Control			Matching criteria	Sample size (AF/control)
		SS	SG	GG	SS	SG	GG		
Lai et al. [7]	2002	7	37	64	18	44	46	Age, sex, BMI, ethnicity	108/108
Ni et al. [10]	2004	3	37	54	10	48	72	Age, sex, ethnicity	94/130
Zeng et al. [8]	2005	10	60	71	23	100	115	Age, sex, ethnicity	141/238
Qu and Yin [9]	2008	6	26	31	15	32	17	Age, sex, ethnicity	63/64
Xu et al. [11]	2008	9	61	77	16	56	75	Age, sex, BMI, ethnicity	147/147
Yao et al. [12]	2011	28	117	158	40	159	129	Age, sex, BMI, ethnicity	303/328

AF: atrial fibrillation; BMI: body mass index.

Polymerase chain reaction-restriction fragment length polymorphism genotyping method and case-control study design have been adopted in all of the above studies.

**Table 2:** Summary of meta-analysis of association of *Mink* S38G gene polymorphism and atrial fibrillation (AF) in the Chinese population.

Genetic model	Pooled OR (95% CI)	*P* value	Study number	AF size	Control size	*P*_heterogeneity(*I*^2^%)_
Allelic genetic model	1.380 (1.200–1.600)	<0.00001*	6	856	1015	0.140 (39.8%)

Recessive genetic model	1.193 (1.033–1.377)	0.017*	6	856	1015	0.057* (53.5%)
Subgroup 1: SG1 < 60	1.320 (0.974–1.789)	0.073	3	265	302	0.055* (65.6%)
Subgroup 2: SG1 ≥ 60	1.162 (1.036–1.302)	0.010*	3	591	713	0.106 (55.4%)

Dominant genetic model	1.057 (1.025–1.089)	<0.00001*	6	856	1015	0.437 (0%)

Additive genetic model	1.105 (1.036–1.178)	0.002*	6	856	1015	0.066* (51.6%)
Subgroup 1: SG1 < 60	1.177 (1.017–1.361)	0.029*	3	265	302	0.038* (69.4%)
Subgroup 2: SG1 ≥ 60	1.078 (1.024–1.135)	0.004*	3	591	713	0.316 (13.1%)

Homozygous genetic model	1.128 (1.068–1.191)	<0.00001*	6	856	1015	0.180 (34.1%)
Heterozygous genetic model	1.078 (1.014–1.146)	0.016*	6	856	1015	0.627 (0.0%)

*P < 0.05.

Abbreviations: AF: atrial fibrillation; CI: confidence interval; OR: odds ratio; AF size: the total number of AF cases; control size: the total number of control group; allelic genetic model: G allele distribution frequency; recessive genetic model: GG versus SG + SS; dominant genetic model: SG + GG versus SS; additive genetic model: total G allele versus total S allele; homozygous genetic model: GG versus SS; heterozygous genetic model: SG versus SS; SG1: SG genotype number of AF group.

**Table 3:** The meta-regression results among 6 studies under the recessive genetic model for the association of *Mink* S38G gene polymorphism with AF in the Chinese population.

	Coefficient	Standard error	*T* value	*P* value	95% confidence interval
SS1	0.0883416	0.0103416	8.54	0.003*	0.05543~0.1212532
SG1	−0.0245643	0.0028102	−8.74	0.003*	−0.0335075~−0.0156211
_cons	0.678298	0.0669228	10.14	0.002*	0.4653198~0.8912762

*P < 0.05.

Coefficient: regression coefficient; the regression coefficients are the estimated increase in the lnOR per unit increase in the covariates; cons: constant item; SS1: SS genotype number of AF group sample size; SG1: SG genotype number of AF group.

**Table 4:** The meta-regression results among 6 studies under the additive genetic model for the association of *Mink* S38G gene polymorphism with AF in the Chinese population.

	Coefficient	Standard error	*T* value	*P* value	95% confidence interval
SS1	0.0387749	0.0047476	8.17	0.004*	0.0236658~0.0538841
SG1	−0.0112773	0.0012901	−8.74	0.003*	−0.015383~−0.0071717
_cons	0.3500916	0.030723	11.40	0.001*	0.2523172~0.4478659

*P < 0.05.

Coefficient: regression coefficient; the regression coefficients are the estimated increase in the lnOR per unit increase in the covariates; cons: constant item; SS1: SS genotype number of AF group sample size; SG1: SG genotype number of AF group sample size.

## References

[1] Go AS, Hylek EM, Phillips KA (2001). Prevalence of diagnosed atrial fibrillation in adults: national implications for rhythm management and stroke prevention: the anticoagulation and risk factors in atrial fibrillation (ATRIA) study. *Journal of the American Medical Association*.

[2] Lip GY, Brechin CM, Lane DA (2012). The global burden of atrial fibrillation and stroke: a systematic review of the epidemiology of atrial fibrillation in regions outside north america and europe. *Chest*.

[3] Wolf PA, Abbott RD, Kannel WB (1991). Atrial fibrillation as an independent risk factor for stroke: the framingham study. *Stroke*.

[4] Wachter R, Lahno R, Haase B (2012). Natriuretic peptides for the detection of paroxysmal atrial fibrillation in patients with cerebral ischemia—the find-AF study. *PLoS ONE*.

[5] Nattel S (2002). New ideas about atrial fibrillation 50 years on. *Nature*.

[6] Nattel S (1999). Atrial electrophysiological remodeling caused by rapid atrial activation: underlying mechanisms and clinical relevance to atrial fibrillation. *Cardiovascular Research*.

[7] Lai L-P, Su M-J, Yeh H-M (2002). Association of the human minK gene 38G allele with atrial fibrillation: evidence of possible genetic control on the pathogenesis of atrial fibrillation. *American Heart Journal*.

[8] Zeng Z-Y, Pu J-L, Tan C (2005). The association of single nucleotide polymorphism of slow delayed rectifier K+ channel genes with atrial fibrillation in Han nationality Chinese. *Chinese Journal of Cardiovascular Diseases*.

[9] Qu ZJ, Yin YH (2008). Correlations between *Mink* S38G polymorphism and atrial fibrillation in old patients with chronic heart failure. *Chinese Journal of Biochemistry and Molecular Biology*.

[10] Ni AZ, Wang R, Liang B, Zhu XX, Lin J (2004). Relevance of gene *KCNE1* and lone atrial fibrillation. *Shanghai Medical Journal*.

[11] Xu L-X, Yang W-Y, Zhang H-Q, Tao Z-H, Duan C-C (2008). Study on the correlation between CETP TaqIB, *KCNE1* S38G and *eNOS* T-786C gene polymorphisms for predisposition and non-valvular atrial fibrillation. *Zhonghua Liuxingbingxue Zazhi*.

[12] Yao J, Ma Y-T, Xie X, Liu F, Chen B-D, An Y (2011). Association of rs1805127 polymorphism of *KCNE1* gene with atrial fibrillation in Uigur population of Xinjiang. *Chinese Journal of Medical Genetics*.

[13] Clark MF, Baudouin SV (2006). A systematic review of the quality of genetic association studies in human sepsis. *Intensive Care Medicine*.

[14] Cochran WG (1968). The effectiveness of adjustment by subclassification in removing bias in observational studies. *Biometrics*.

[15] DerSimonian R, Laird N (1986). Meta-analysis in clinical trials. *Controlled Clinical Trials*.

[16] Mantel N, Haenszel W (1959). Statistical aspects of the analysis of data from retrospective studies of disease. *Journal of the National Cancer Institute*.

[17] Egger M, Smith GD, Schneider M, Minder C (1997). Bias in meta-analysis detected by a simple, graphical test. *British Medical Journal*.

[18] Ioannidis JPA, Boffetta P, Little J (2008). Assessment of cumulative evidence on genetic associations: Interim guidelines. *International Journal of Epidemiology*.

[19] Fatini C, Sticchi E, Genuardi M (2006). Analysis of *minK* and *eNOS* genes as candidate loci for predisposition to non-valvular atrial fibrillation. *European Heart Journal*.

[20] Šmalcelj A, Sertić J, Golubić K, Jurčić L, Banfić L, Brida M (2009). Interactions of *MinK* and *e-NOS* gene polymorphisms appear to be inconsistent predictors of atrial fibrillation propensity, but long alleles of *ESR1* promoter TA repeat may be a promising marker. *Collegium Antropologicum*.

[21] Sinner MF, Ellinor PT, Meitinger T, Benjamin EJ, Kääb S (2011). Genome-wide association studies of atrial fibrillation: past, present, and future. *Cardiovascular Research*.

[22] Yang Y, Xia M, Jin Q (2004). Identification of a *KCNE2* gain-of-function mutation in patients with familial atrial fibrillation. *American Journal of Human Genetics*.

[23] Barhanin J, Lesage F, Guillemare E, Fink M, Lazdunski M, Romey G (1996). K_v_ LQT1 and IsK (minK) proteins associate to form the *I*
_KS_ cardiac potassium current. *Nature*.

[24] Schulze-Bahr E, Wang Q, Wedekind H (1997). *KCNE1* mutations cause jervell and Lange-Nielsen syndrome. *Nature genetics*.

[25] Splawski I, Shen J, Timothy KW (2000). Spectrum of mutations in Long-QT Syndrome genes: *KVLQT1*, *HERG*, *SCN5A*, *KCNE1*, and *KCNE2*. *Circulation*.

[26] Paulussen ADC, Gilissen RAHJ, Armstrong M (2004). Genetic variations of *HCNQ1*, *KCNH2*, *SCN5A*, *KCNE1*, and *KCNE2* in drug-induced long QT syndrome patients. *Journal of Molecular Medicine*.

[27] Chevillard C, Attali B, Lesage F (1993). Localization of a potassium channel gene (KCNE1) to 21q22.1-q22.2 by *in situ* hybridization and somatic cell hybridization. *Genomics*.

[28] Chen Y-H, Xu S-J, Bendahhou S (2003). *KCNQ1* gain-of-function mutation in familial atrial fibrillation. *Science*.

[29] Lin Z, Li Z, Yafei L, Xiangyu M (2009). MinK gene G112A polymorphisms and atrial fibrillation: a Meta-analysis. *Journal of Medical Colleges of PLA*.

[30] Prystupa A, Dzida G, Myśliński W, Małaj G, Lorenc T (2006). MinK gene polymorphism in the pathogenesis of lone atrial fibrillation. *Kardiologia Polska*.

[31] Lou S, Lu L, Wu LQ, Shen WF, JinQ (2006). Association between human potassium channel *β*-subunit gene KCNE1-S38G polymorphism and atrial fibrillation. *Journal of Diagnostics Concepts & Practice*.

